# Expression Patterns of TOP2A and SIRT1 Are Predictive of Survival in Patients with High-Risk Soft Tissue Sarcomas Treated with a Neoadjuvant Anthracycline-Based Chemotherapy

**DOI:** 10.3390/cancers13194877

**Published:** 2021-09-29

**Authors:** Luc M. Berclaz, Annelore Altendorf-Hofmann, Hans Roland Dürr, Alexander Klein, Martin K. Angele, Markus Albertsmeier, Nina-Sophie Schmidt-Hegemann, Dorit Di Gioia, Thomas Knösel, Lars H. Lindner

**Affiliations:** 1 Department of Internal Medicine III, University Hospital, Ludwig-Maximilians-University (LMU) Munich, Marchioninistr. 15, 81377 Munich, Germany; dorit.digioia@med.uni-muenchen.de (D.D.G.); Lars.Lindner@med.uni-muenchen.de (L.H.L.); 2 Department of General, Visceral and Vascular Surgery, Friedrich-Schiller University Jena, Am Klinikum 1, 07743 Jena, Germany; Annelore.altendorf-hofmann@gmx.de; 3 Musculoskeletal Oncology, Department of Orthopaedic Surgery, Physical Medicine and Rehabilitation, University Hospital, Ludwig-Maximilians-University (LMU) Munich, Marchioninistr. 15, 81377 Munich, Germany; hans_roland.duerr@med.uni-muenchen.de (H.R.D.); alexander.klein@med.uni-muenchen.de (A.K.); 4 Department of General, Visceral and Transplantation Surgery, University Hospital, Ludwig-Maximilians-University (LMU) Munich, Marchioninistr. 15, 81377 Munich, Germany; martin.angele@med.uni-muenchen.de (M.K.A.); Markus.Albertsmeier@med.uni-muenchen.de (M.A.); 5 Department of Radiation Oncology, University Hospital, Ludwig-Maximilians-University (LMU) Munich, Marchioninistr. 15, 81377 Munich, Germany; Nina-Sophie.Hegemann@med.uni-muenchen.de; 6 LMU Institute of Pathology, Ludwig-Maximilians-University (LMU) Munich, Thalkirchner Str. 36, 80337 Munich, Germany; Thomas.Knoesel@med.uni-muenchen.de

**Keywords:** soft tissue sarcoma, TOP2A, SIRT1, tissue microarray, immunohistochemistry, biomarker, precision oncology

## Abstract

**Simple Summary:**

High-risk soft tissue sarcomas (HR-STS) account for less than 1% of all malignancies in adults. Despite optimal local treatment, almost half of patients will die within five years of their diagnosis. Chemoresistance is a major responsible mechanism for treatment failure in advanced tumor stages. In contrast to other cancer types, molecular predictors of response to chemotherapy and survival have not been identified and put into clinical practice by now. We analyzed the predictive value of two molecules involved in the working and resistance mechanisms to doxorubicin, TOP2A and SIRT1, in a large cohort of locally advanced HR-STS who underwent a neoadjuvant anthracycline-based chemotherapy with a long-term follow-up. Our results show sarcoma subtype-specific patterns of TOP2A and SIRT1 expression. We demonstrate significant differences in overall survival according to the TOP2A and SIRT1 expression status. Both markers can be used as clinically significant predictive indicators for HR-STS patients scheduled for neoadjuvant anthracycline-based chemotherapy.

**Abstract:**

Molecular predictors of response to chemotherapy and survival have not been put into clinical practice in high-risk soft tissue sarcomas (HR-STS) by now. The expression of TOP2A and SIRT1 has implications for the mechanism of action of doxorubicin, which is the backbone of chemotherapy in HR-STS. Pre-treatment samples of 167 patients with HR-STS were collected. Protein expression levels of TOP2A and SIRT1 were evaluated with tissue microarrays and immunohistochemistry and correlated with clinicopathological parameters, including overall survival (OS). The expression of TOP2A and SIRT1 was seen in 47% and 60% of patients with HR-STS, respectively. TOP2A expression was associated with higher tumor grading and shorter 5-year OS. The expression of SIRT1 was correlated with a better 5- and 10-year OS. The combination of high SIRT1 and low TOP2A (“Top survivors”) significantly predicted a better OS compared to other biomarker combinations. A multivariate analysis confirmed the expression of SIRT1 and the “Top survivor” biomarker combination as independent predictive factors of OS. This is the first study to associate SIRT1 overexpression with a statistically significant prolongation of OS in HR-STS. Both individual markers and their combination can be used as predictive indicators for HR-STS patients scheduled for neoadjuvant anthracycline-based chemotherapy.

## 1. Introduction

High-risk soft tissue sarcomas (HR-STS) are defined by a size of 5 cm or more, deep localization in relation to the fascia and grade two or three according to the French Fédération Nationale de la Lutte Contre le Cancer (FNCLCC) grading system [1]. They have a high metastatic potential and mostly metastasize hematogenously with the lung as the main target organ [2]. Despite optimal local treatment, almost half of patients with HR-STS will die within 5 years of their diagnosis [3,4]. Drug resistance is believed to cause treatment failure in over 90% of patients with metastatic cancer [5].

Despite the current knowledge about biological and clinical differences in sarcoma subtypes, patients have mostly been treated in a “one-size-fits-all” approach with anthracyclines and alkylating agents for the past 3 decades [6,7,8,9,10]. Broadly usable, clinically significant biomarkers that predict therapy response and survival have yet to be discovered in sarcomas. In other tumors, biomarkers with prognostic and predictive importance have already been found. An important example is the use of hormone receptors in breast cancer [11]. Rare examples in sarcomas are the use of AMPD2 in undifferentiated pleomorphic sarcomas or PD-L1 in soft tissue sarcomas, which were correlated with survival by Orth et al., or the MDM2 amplification status in liposarcomas, which correlated with drug sensitivity and clinical outcomes in a study by Bill et al. [12,13,14]. They have no use in clinical routine at present. Prognostic factors currently rely on clinical evaluations, such as TNM staging, topography, tumor size and the assessment of surgical margins [15,16]. The lack of molecular predictors leads to a fraction of patients suffering from side effects of chemotherapy without achieving any benefit, as sensitivity to chemotherapeutic agents is not routinely tested despite the common presence of intrinsic and acquired chemoresistance [5]. With novel biomarkers, future treatment plans could be adjusted to the individual patient. In addition, signaling pathways and molecular mechanisms could be specifically targeted to minimize chemoresistance and improve prognosis.

The aim of this study was to analyze expression patterns of two molecular markers involved in the working mechanisms and resistance pathways of doxorubicin: TOP2A, which regulates the DNA structure and cell proliferation and acts as the main target of doxorubicin, and SIRT1, an NAD-dependent histone deacetylase which regulates cellular differentiation and responses to apoptosis, stress and DNA damage.

Variations in the expression of TOP2A have been associated with chemoresistance and changes in outcome. The predictive properties of TOP2A have been described in several studies, with varying results: The overexpression of TOP2A was linked to a poor prognosis and unfavorable five-year overall survival (OS) in different soft tissue sarcomas [17,18,19]. In leiomyosarcomas, TOP2A was highly expressed but failed to predict outcomes [20]. TOP2A overexpression was also correlated with a better pathohistological response and a decreased risk of relapse in locally advanced soft tissue sarcomas [21]. In bladder cancer, TOP2A downregulation was predictive for a poor response to neoadjuvant chemotherapy, making it one of fourteen predictive genes among over 27,000 genes in a genome-wide microarray assay [22].

Depending on the expression status and cellular context, SIRT1 acts as a tumor suppressor or as an oncogene and resistance mechanism to doxorubicin. In gastric cancers, SIRT1 was associated with lymphatic invasion, vessel invasion, lymph node metastasis and a poor cancer-specific survival [23]. SIRT1 was expressed in 71% of 104 sarcoma patients in a study by Kim et al., regardless of the histological subtype. SIRT1 was also associated with advanced clinicopathological parameters, including stage, grade and metastasis. In addition, event-free survival and OS were significantly reduced with high SIRT1 levels [24]. The importance of SIRT1 in carcinogenesis was also discussed in a study by Chu et al. SIRT1 was upregulated in five different cell lines, including osteosarcoma. Chemoresistance was observed in all cell types, independent of the used chemotherapeutic regimen. In addition, SIRT1 was responsible for the overexpression of other proteins responsible for drug resistance, including MDR1. Chemoresistance was then reversed by selectively inhibiting SIRT1 with small interfering RNAs (SiRNAs), enhancing the response to chemotherapy in the drug-resistant cells by an additional 25% to 30% [25].

In the present study, we analyze the expression of TOP2A and SIRT1 in a large and well-characterized cohort of high-risk soft tissue sarcoma patients with a long-term follow-up and correlate our findings with clinical tumor characteristics and survival data. We show that TOP2A and SIRT1 display distinct expression patterns in different STS subtypes. TOP2A and SIRT1 expression levels are inversely correlated to patient survival. In a multivariate analysis, the expression of SIRT1 and the biomarker combination of high SIRT1 and low TOP2A (“Top survivors”) are confirmed as independent predictive factors of OS.

## 2. Results

### 2.1. Patient Cohort

The baseline characteristics of the investigated cohort are summarized in Table 1.

In total, 167 patients were analyzed. Median age was 53 years (18–79 years), 83 patients were female and 84 were male. The most common sarcoma subtypes were undifferentiated pleomorphic sarcomas (UPS, 33%), followed by liposarcomas (21%). Half of the tumors were high grade (G3, 50%). A total of 65% of all patients were diagnosed with non-extremity sarcomas. Most patients (84%) underwent a R0 or R1 resection of their primary tumor. Most patients had a stable disease (SD) according to the RECIST tumor response criteria (47%). In total, 100 patients expressed a high level of SIRT1 (60%). TOP2A was positive in 79 patients (47%). We correlated the expression of TOP2A and SIRT1 with all clinical factors in Table 1. Of those parameters, the correlation of TOP2A and grading was statistically significant (G2 vs. G3, *p* = 0.001, Fisher’s exact two-sided test) (Table 2). The expression of SIRT1 did not significantly correlate with a higher FNCLCC tumor grading (G2 vs. G3, *p* = 0.115) (Table 2). The correlation of TOP2A and SIRT1 expression with AJCC staging was not statistically significant (Stage II vs. III, *p* = 0.688 and *p* = 0.417) (Table 2). The correlation of tumor response and TOP2A expression was not statistically significant (*p* = 0.184). In addition, the association between tumor response and SIRT1 expression was not statistically significant (*p* = 0.940). Lastly, the correlation between high TOP2A and high SIRT1 expression was statistically significant (*p* = 0.001).

### 2.2. TOP2A and SIRT1 Expression in Sarcoma Subtypes

**Figure 1:**
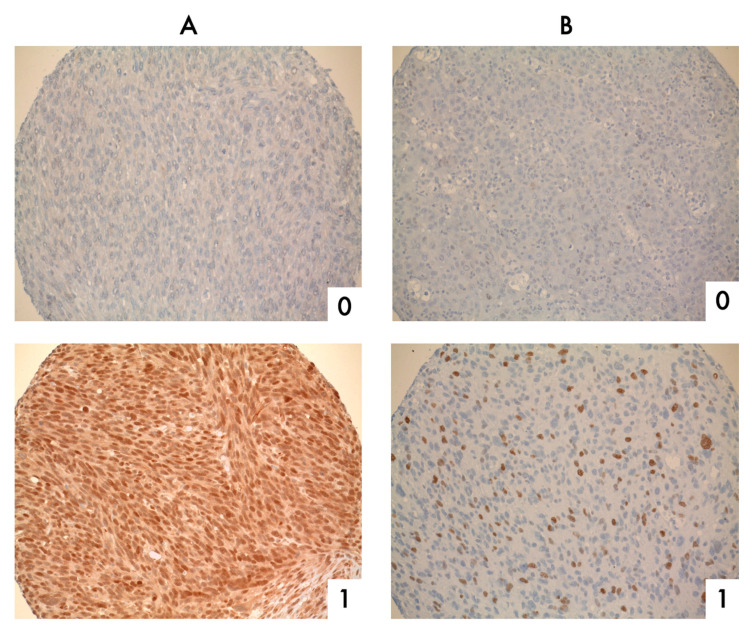
Stained tissue microarray cores. Representative micrographs of cores on a tissue microarray stained for (**A**) SIRT1 and (**B**) TOP2A. Numbers represent semiquantitative scoring of immunostaining: 0, negative; 1, positive. Magnification 20×.

The expression of both biomarkers varied between different sarcoma subtypes (Table 2). Malignant peripheral nerve sheath tumors and synovial sarcomas were strongly correlated with TOP2A expression (67% and 65%, respectively). SIRT1 was mostly expressed in synovial sarcomas and undifferentiated pleomorphic sarcomas (75% and 67%). Examples of immunohistochemistry staining for TOP2A and SIRT1 are shown in Figure 1. 

### 2.3. Study Population Survival Analysis

The median follow-up time was 120 months. All but nine patients (95%) were followed until the end of 2020 or until death. The nine remaining patients were treated in Germany and returned to their home country after treatment, which explains the loss of a follow-up. We performed a univariate survival analysis with all clinical parameters mentioned in Table 1. Age (<55 y median OS >120 months vs. >55 y median OS 52 months, *p* = 0.033) and surgical margins (R0/R1 median OS 114 months vs. R2/no resection median OS 19 months, *p* < 0.001) were significant predictors of OS.

**Figure 2:**
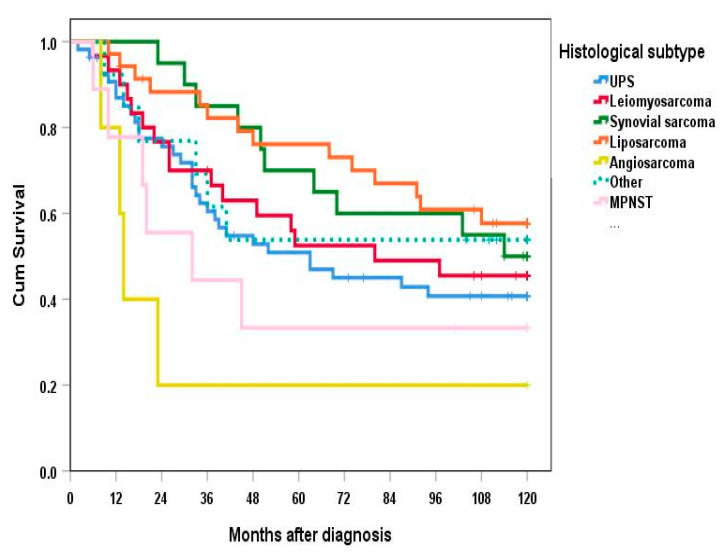
Overall survival according to histological subtype. UPS—undifferentiated pleomorphic sarcoma. MPNST—malignant peripheral nerve sheath tumor. Other—3 Chondrosarcomas, 4 Myxofibrosarcomas, 1 Alveolar sarcoma, 2 Carcinosarcomas, 1 Rhabdomyosarcoma, 2 malignant solitary fibrous tumors.

OS was significantly worse in angiosarcomas compared to all histologies except undifferentiated pleomorphic sarcomas (*p* < 0.05) (Figure 2). In addition, OS was significantly worse in malignant peripheral nerve sheath tumors compared to liposarcomas (*p* = 0.043). Five- and ten-year OS was not calculated in the “Other” group due to major differences in sarcoma subtypes and a low case number.

Statistically nonsignificant predictors of OS were sex (male median OS 68 months vs. female median OS > 120 months, *p* = 0.084), grading (G2 median OS > 120 months vs. G3 median OS 52 months, *p* = 0.108), tumor location (extremity median OS > 120 months vs. non-extremity median OS 80 months, *p* = 0.222) and tumor size (<80 mm median OS 114 months vs. >80 mm median OS 68 months, *p* = 0.224). 

### 2.4. Correlation of TOP2A and SIRT1 Expression with Overall Survival

**Figure 3:**
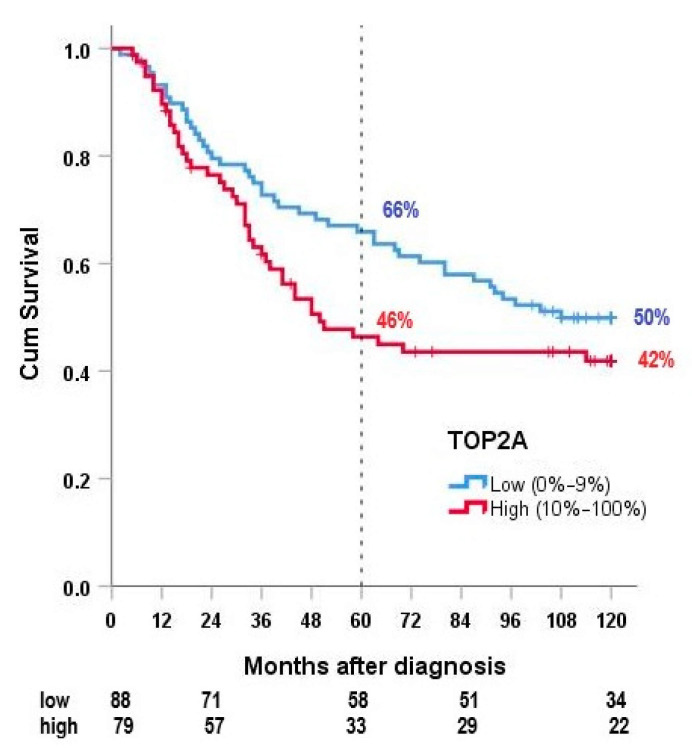
Overall survival according to TOP2A expression.

In this study, high TOP2A expression was correlated with a worse prognosis in HR-STS (Figure 3). Median OS was 50 months compared to 108 months in TOP2A-negative tumors, 5-year OS 46% vs. 66% and 10-year OS 42% compared to 50%. The difference in the 5-year OS was statistically significant (*p* = 0.039). In contrast, the difference in the 10-year OS was not statistically significant (*p* = 0.160).

When analyzing the effect of TOP2A expression on the four most important histological subgroups, differences in OS were only statistically significant in synovial sarcomas (64 months vs. >120 months, *p* = 0.021).

**Figure 4:**
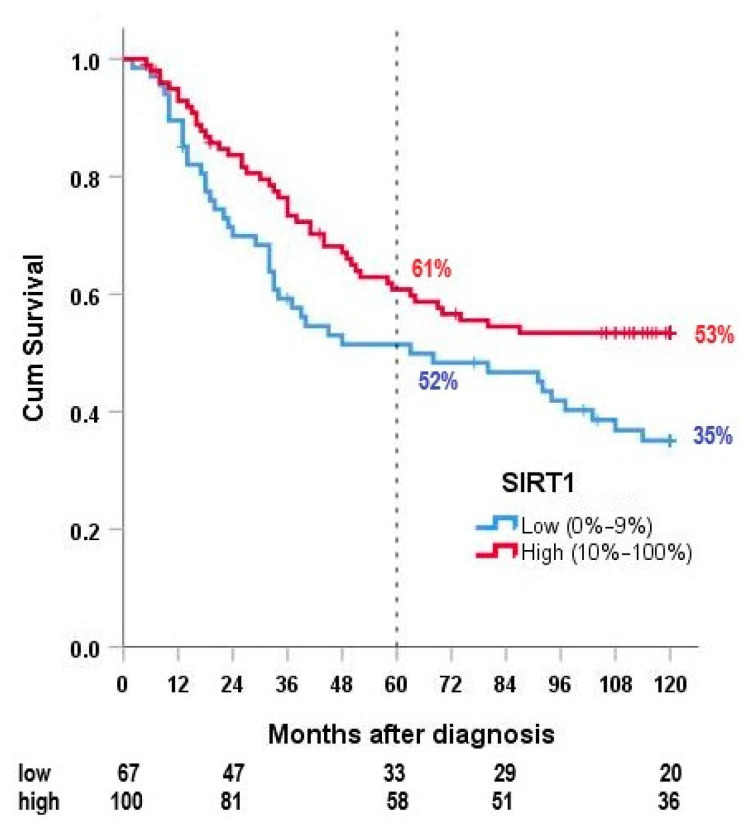
Overall survival according to SIRT1 expression.

The high expression of SIRT1 was correlated with a longer OS in HR-STS (Figure 4). Median OS was >120 months in patients with a high expression of SIRT1 compared to 63 months with low SIRT1 levels. Differences in 5- and 10-year OS were also statistically significant (5-year OS 61% vs. 52%, 10-year OS 53% vs. 35%, *p* = 0.025). Differences in OS in the four most important histological subgroups were not statistically significant.

**Figure 5:**
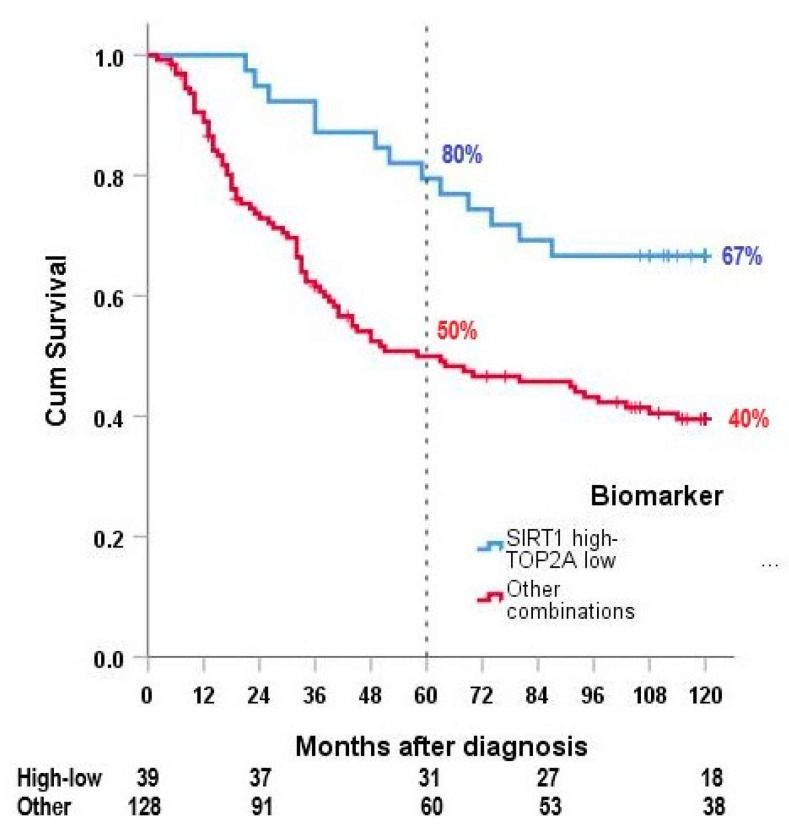
Overall survival with favorable “Top Survivor” biomarker expression (high SIRT1, low TOP2A) compared to other biomarker combinations.

In this study, 39 patients (23%) were diagnosed with HR-STS expressing both high SIRT1 and low TOP2A levels. As mentioned before, high SIRT1 and low TOP2A levels favored OS in this patient cohort. This specific “Top survivor” biomarker combination had a significant positive impact on OS (5-year OS 80% vs. 50%, 10-year OS 67% vs. 40%, *p* = 0.002) (Figure 5).

### 2.5. TOP2A and SIRT1 in Multivariate Analysis

To control for confounding factors, a multivariate analysis was performed with five previously analyzed prognostic factors (age, surgical resection margins, grade, TOP2A and SIRT1 expression) using a Cox proportional hazards model. The low expression of SIRT1 and high expression of TOP2A were independent predictors of shorter OS. Radical resection, younger age (<55 years) and lower tumor grade were associated with a longer OS in HR-STS. All factors except grade and TOP2A expression proved to be statistically significant (Table 3).

Secondly, we performed a multivariate analysis with several clinical variables (age, resection margins, grade) and the favorable “Top survivor” combination of high SIRT1 and low TOP2A expression, which was previously correlated with a significant survival benefit. In this setting, a combination of high SIRT1 and low TOP2A expression proved to be an independent statistically significant predictor of OS compared to other biomarker combinations (*p* = 0.002). Patients with other biomarker combinations were 2.547 times more at risk to suffer from a shorter OS (Table 4).

## 3. Discussion

In this study, we analyzed the expression of TOP2A and SIRT1 in a well-characterized cohort of high-grade soft tissue sarcoma patients using tissue microarrays and immunohistochemistry. These markers were chosen because of their relationship to doxorubicin and chemoresistance. Our findings suggest that patients with high TOP2A expressing tumors have a shorter OS than patients with low TOP2A levels. In contrast, a high expression of SIRT1 correlates with a prolonged OS. In addition to these findings, patients with a combination of high SIRT1 and low TOP2A expression (“Top survivors”) had a significantly longer OS than patients with other biomarker combinations.

Some classically significant clinical predictors of OS, including grading, tumor size and tumor location, did not reach statistical significance in our study population. This was partly due to the sample size and large number of high-risk soft tissue sarcoma subgroups. Nevertheless, absolute numbers correlated with previous studies on this subject (grading: G2 median OS > 120 months vs. G3 median OS 52 months, *p* = 0.108; tumor location: extremity median OS > 120 months vs. non-extremity median OS 80 months, *p* = 0.222; tumor size: <80 mm median OS 114 months vs. >80 mm median OS 68 months, *p* = 0.224).

Our results indicated that a high TOP2A expression leads to a shorter 5-year OS in patients with HR-STS (5-year OS 46% vs. 66%, *p* = 0.039). These findings correlated with current literature about TOP2A expression and cancer prognosis; TOP2A expression was already described as an independent predictor of an unfavorable prognosis in sarcomas in a study conducted by Da Cunha et al. in 2012. They successfully included TOP2A in a prognostic scoring system along with the histologic grade, surgical margins and tumor size [17]. Our results correlated with these findings. A different mix of histological subtypes and other inclusion criteria between their study and ours (only high-risk patients (>5 cm, G2/G3, deep to the fascia), no evidence of metastases, different biomarker scoring evaluations) did not change the prognostic use of TOP2A. 

Skotheim et al. correlated the TOP2A gene and protein overexpression with a poor 10-year OS in malignant peripheral nerve sheath tumors (MPNST) [19]. Our results also indicated that TOP2A expression predicts a poor survival in MPNST. However, as this study only included 9 MPNST, our results were not statistically significant. In general, the correlation between TOP2A expression and a shorter OS was not statistically significant in our subgroups partly due to the low sample sizes. This underlines the difficulty with research on HR-STS: A statistically significant analysis of histological subgroups requires large patient cohorts, which is only possible in large-volume sarcoma centers over a long period of time.

Interestingly, our results indicated that TOP2A expression only correlated with a shorter 5-year OS compared to a 10-year OS (*p* = 0.039 vs. *p* = 0.160). This might be due to the fact that many patients with a low TOP2A expression were censored in the follow-up period between five and ten years, which limited the significance of the 10-year OS in this regard.

Molecular mechanisms associated with TOP2A overexpression and tumor progression or chemoresistance still need to be established. Possible explanations are an increase in TOP2A mutations leading to doxorubicin insensitivity and a decreased apoptosis signaling in affected cells [26,27]. Another molecular explanation is TOP2A deregulation caused by YB-1 overexpression. YB-1 is a DNA-binding protein which is located on the promoter of the TOP2A gene. YB-1 overexpression results in downstream TOP2A overexpression and tumor progression. Oda et al. demonstrated a shorter OS in synovial sarcomas due to YB-1 overexpression [18]. This example demonstrates the fragility of regulatory mechanisms in sarcomas, as the over- and underexpression of other molecules can also affect downstream TOP2A activity and result in chemoresistance and poor prognosis. 

This study identified SIRT1 as an independent predictor of OS in HR-STS. High SIRT1 expression correlated with a better 5- and 10-year OS (10-year OS 53% vs. 35%, *p* = 0.025). Our multivariate analysis suggested that the prognostic value of SIRT1 is robust, even in relation to previously established clinical parameters such as surgical margins or patient age. We did not expect these results. Kim et al. correlated SIRT1 overexpression with advanced clinicopathological parameters, reduced event-free survival (EFS) and reduced OS in soft tissue sarcoma patients [24]. Additionally, the selective inhibition of SIRT1 lead to a reduction in chemoresistance in osteosarcoma cell lines in an experimental study by Chu et al. [25]. These findings were consistent with the results of Ma et al.; in their study, a selective inhibition of SIRT1 and SIRT2 impaired the survival of pediatric rhabdomyosarcoma and synovial sarcoma cell lines [28]. The correlation between SIRT1 overexpression and poor survival was explained through the deacetylation of downstream molecules of apoptosis, which impairs a physiological DNA damage response, and transcriptional inactivity of tumor suppressor genes [29,30]. Another study explained that SIRT1 supports tumor development by making the tumor “addicted” to sirtuins [31]. 

Only a few studies correlate with our findings on a better survival with SIRT1 expression. These studies were conducted on other cancer types, including breast, ovarian and colon cancer, which limits their comparison to our results [32,33]. A possible explanation for our results could be the dual role of sirtuins in cancer as described by Bosch-Presegué et al. It is still not clear why SIRT1 acts as a tumor suppressor and as an oncogene depending on the situation in the individual cell. They postulated that SIRT1 may swap from a tumor suppressor to oncogene after reaching a certain stress threshold [31]. This model usually distinguishes healthy cells from tumor cells, and future research should investigate SIRT1 specifically in HR-STS to determine if there is a dual role specifically in malignant sarcoma cells. Lastly, as our patient population was unique in regard to the use of hyperthermia during treatment, the effect of hyperthermia on sirtuin expression and vice versa could be further analyzed in vitro.

We identified a subgroup of patients with HR-STS that distinguished themselves from other patients according to their TOP2A and SIRT1 expression status (“Top survivors”). Patients with high SIRT1 and low TOP2A expression had a statistically significant improvement of OS compared to other biomarker combinations (10-year OS 67% vs. 40%, *p* = 0.002). A multivariate analysis proved this combination to be an independent statistically significant predictor of OS (*p* = 0.002). This is the first study to identify such a predictive model. If the predictive property of our biomarker combination remains robust in future studies, it could be used as an additional tool in individual therapy decisions.

Strengths of this study were the analysis of TOP2A and SIRT1 on a large patient cohort of high-grade soft tissue sarcomas. Sarcomas are very rare tumors, a cohort of 167 patients was, therefore, considered large in this tumor entity. In addition, our inclusion criteria (only high-risk sarcomas, no evidence of metastases, only pre-treatment biopsies) caused our population to be more homogenous. The follow-up of more than ten years was another strength of this study.

A limitation of this study was the chosen cutoff for a positive vs. negative biomarker expression (0–9% vs. 10–100%). The cutoff value varies between studies and should be standardized to better compare results, as there is no consensus about the definition of a “high” and “low” expression of TOP2A and SIRT1. Soto Rodrigo et al. used the median percentage of TOP2A expression as a cutoff in their sarcoma cohort, which could be a clever way to distinguish high from low expression [21]. Another limitation of this study was the correlation of chemotherapy response with survival rates. We chose biomarkers specifically related to chemoresistance and used OS as a measurable outcome in our study population. Future studies could include other variables to measure the response to chemotherapy. An example is the Salzer-Kuntschik tumor regression grading system used in osteosarcomas [34]: tumor samples are analyzed after chemotherapy and the degree of tumor necrosis is taken as a surrogate for the response to chemotherapy. High rates of necrosis are, therefore, correlated with a good histopathologic response to chemotherapy. In a second study, these findings were correlated with a better 10-year overall and event-free survival [35]. Such a grading system could be interesting for our patient cohort. Lastly, the prognostic value of TOP2A and SIRT1 in specific histological subtypes (compared to HR-STS in general) was limited due to the limited number of specific patient cases.

Future studies should compare the expression of TOP2A and SIRT1 in pre- and post-chemotherapy samples. This could lead to new insights in the adaptive properties of chemotherapy resistance in HR-STS. The predictive properties of TOP2A and SIRT1 could also be examined in patients with HR-STS that were not treated with chemotherapy, working as a control group. In addition, future studies could analyze TOP2A and SIRT1 on a genomic level and compare these results to the protein level of the same markers, as some studies described differences in expression between genome and proteome. 

## 4. Materials and Methods

### 4.1. Patient Selection

An exploratory retrospective cohort study design was chosen to address the research question. Eligible patients had proven soft tissue sarcoma with the following risk criteria: tumor diameter 5 cm or larger, French Fédération Nationale des Centres de Lutte Contre le Cancer (FNCLCC) grade 2 or 3, deep to the fascia, and no evidence of distant metastases. *n* = 167 patients treated between 1989 and 2012 were included in this study. Data on clinical parameters were extracted from original clinical and pathology reports at the LMU University Hospital of Munich, Germany. Clinical data were updated until February 2021.

### 4.2. Procedures

All patients with HR-STS were to receive a multimodal treatment, including neoadjuvant doxorubicin-based chemotherapy, surgery, radiation therapy and adjuvant doxorubicin-based chemotherapy. Chemotherapy was combined with regional hyperthermia (RHT). In the end, nearly all patients underwent surgical resection of the tumor and about two-thirds received radiotherapy.

Neoadjuvant and adjuvant chemotherapy was given in 3-week intervals consisting of doxorubicin, ifosfamide, and, partly, etoposide. Etoposide was eliminated from chemotherapy protocols after 2010. In the etoposide-containing regimen (EIA), 50 mg/m^2^ of doxorubicin was given on day 1, combined with 1500 mg/m^2^ of ifosfamide on days 1 to 4 and 125 mg/m^2^ of etoposide on days 1 and 3. In the regimen without etoposide (AI), 60 mg/m^2^ of doxorubicin was given in combination with 3000 mg/m^2^ of ifosfamide on days 1 to 3 or 1500 mg/m^2^ on days 1 to 4 (AI60/9 vs. AI60/6).

Chemotherapy was given concurrently with regional hyperthermia (42 °C for a 60 min period on day 1 and 4 of each cycle). Response to neoadjuvant therapy was evaluated by computed tomography or magnetic resonance imaging and chest radiography after two cycles of induction therapy. Definitive surgery occurred within 4 to 6 weeks of neoadjuvant therapy.

Adjuvant treatment was started within six weeks of local therapy. It consisted of external beam radiation therapy (administered dose between 50.0 and 60.0 Gray, with daily fractions of 1.8 to 2.2 Gray, and a boost up to 66.0 Gray) and another four cycles of adjuvant chemotherapy with regional hyperthermia within 6 weeks of local therapy. Quality of hyperthermia was ensured by current guidelines of the European Society for Hyperthermic Oncology (ESHO) [36,37]. Treatment continued unless progressive disease or unacceptable toxic effects occurred.

### 4.3. Histopathology and Tissue Microarray Construction

Tumor samples originated from tumor biopsies that were taken before the initiation of neoadjuvant treatment at the Ludwig-Maximilians-University hospitals, Munich. In addition to the original pathology reports, microscopic findings (tumor type according to current WHO classifications and degree of differentiation) were reassessed. For tissue microarray (TMA) assembly, representative tumor areas were marked on H&E-stained slides of formalin-fixed, paraffin-embedded tumor samples from all patients according to standard procedures and two 0.6 mm punch biopsies were taken from each sample [38]. Normal tonsillar tissue samples were used as controls on the TMA. In the end, 4 tissue microarrays containing 167 pre-treatment tumor samples from 167 patients with high-grade soft tissue sarcomas were constructed.

### 4.4. Immunohistochemistry

For the immunohistochemical detection of TOP2A and SIRT1, commercially available and validated monoclonal antibodies were used [23,39] (Table 5). Antigen retrieval was carried out by heat treatment with Target Retrieval Solution Citrate (Agilent Technologies, Santa Clara, CA, USA). Staining was performed on a Ventana Benchmark XT Autostainer (Ventana Medical Systems, Tucson, AZ, USA) with a DAB+ Kit (Agilent Technologies, Santa Clara, CA, USA). All slides were counterstained with hematoxylin (Vector Laboratories, Burlingame, CA, USA). An ImmPRESS Anti-Rabbit IgG Polymer Kit was used for detection (Vector Laboratories, Burlingame, CA, USA). To exclude unspecific staining, system controls were included. Tonsillar tissue served as a positive control for immunohistochemistry. Immunostaining of cells was evaluated and scored semi-quantitatively (0 = 0–9%, negative; 1 = 10–100%, positive) (Table 6). All immunohistochemical and pathologic evaluations were carried out independently and blinded together with an experienced pathologist with special expertise in sarcoma pathology (T.K.). In the case of discrepancy, the slides were reevaluated under a multiheaded microscope and consensus reached.

### 4.5. Statistical Analysis

Categorical variables were tested for independence using the Chi square test. Survival was calculated from the date when sarcoma was first diagnosed. OS (patients’ death without regarding the cause of death) was used as the endpoint for estimating prognosis. Survival curves were created using the Kaplan–Meier method, and the log-rank test was used to assess differences in survival. 

Significant and independent predictors of OS were identified by Cox proportional hazard analysis. The forward stepwise procedure was set to a threshold of 0.05. All statistical analyses were performed using SPSS 26.0 (IBM, Chicago, IL, USA) software. Statistical significance was defined as a *p* value < 0.05 for all analyses. 

## 5. Conclusions

TOP2A and SIRT1 showed distinct expression patterns in different high-risk soft tissue sarcoma subtypes. This study confirmed previous results on TOP2A overexpression and shorter OS in HR-STS. It is the first study to associate SIRT1 overexpression with a statistically significant prolongation of OS in HR-STS. Both markers can be used as clinically significant predictive indicators for HR-STS patients scheduled for neoadjuvant anthracycline-based chemotherapy. If the predictive “Top survivor” biomarker combination (high SIRT1, low TOP2A) remains robust in future studies, it could become an additional tool in individual therapy decisions.

## Figures and Tables

**Table 1:** Patient characteristics.

Factor	Strata	*n*	%
Total		167	100
Sex	Male	84	50
	Female	83	50
Histological subtype	Undifferentiated pleomorphic sarcoma	55	33
	Leiomyosarcoma	30	18
	Synovial sarcoma	20	12
	Liposarcoma	35	21
	Angiosarcoma	5	3
	MPNST	9	5
	Other	13	8
Grading	Intermediate (G2)	84	50
	High (G3)	83	50
Location	Extremities	58	35
	Non-Extremities	109	65
Size	50–80 mm	56	33
	≥80 mm	93	56
	Missing	18	11
Surgical margins	R0	44	26
	R1	96	58
	R2 or no resection	27	16
AJCC stage	Stage II	31	18
	Stage III	118	71
	Missing	18	11
Tumor response	Complete/partial response	24	14
	Stable disease	78	47
	Progressive disease	24	14
	Missing	41	25
TOP2A expression	Low (0–9%)	88	53
	High (10–100%)	79	47
SIRT1 expression	Low (0–9%)	67	40
	High (10–100%)	100	60

**Table 2:** Candidate biomarker TOP2A and SIRT1 expression according to histological subtype, grading and AJCC stage. The table shows the number and percentage of patients with high TOP2A and SIRT1 expression.

Histological Subtype	Total	TOP2A		SIRT1	
	*n*	*n*	%	*n*	%
UPS	55	31	56	37	67
Leiomyosarcoma	30	12	40	17	57
Synovial Sarcoma	20	13	65	15	75
Liposarcoma	35	11	31	19	54
Angiosarcoma	5	2	40	3	60
MPNST	9	6	67	3	33
Other	13	4	31	6	46
**Grading**	***n***	***n***	**%**	***n***	**%**
G2	84	28	33	45	54
G3	83	51	61	55	66
**AJCC Stage**	***n***	***n***	**%**	***n***	**%**
Stage II	31	16	52	21	68
Stage III	118	55	47	70	59

PS—undifferentiated pleomorphic sarcoma; MPNST—malignant peripheral nerve sheath tumor. Other—3 Chondrosarcomas, 4 Myxofibrosarcomas, 1 Alveolar sarcoma, 2 Carcinosarcomas, 1 Rhabdomyosarcoma, 2 malignant SFT (solitary fibrous tumor).

**Table 3:** Multivariate analysis of overall survival 1. A Cox proportional hazards model for overall survival was calculated, including our biomarkers TOP2A and SIRT1 and statistically significant clinical parameters.

Factor	Strata	Significance	Hazard Ratio	95.0% CI
Surgical margins	R0/R1 vs. R2 or no resection	0.0001	2.735	(1.632–4.581)
Age		0.020	1.018	(1.003–1.033)
Grade	G2 vs. G3	0.136	1.409	(0.897–2.213)
TOP2A	Low vs. high	0.058	1.566	(0.985–2.489)
SIRT1	High vs. low	0.004	1.935	(1.240–3.018)

**Table 4:** Multivariate analysis of overall survival 2. A Cox proportional hazards model for overall survival was calculated, including the favorable “Top Survivor” biomarker combination (low TOP2A and high SIRT1) and statistically significant clinical parameters.

Factor	Strata	Significance	Hazard Ratio	95.0% CI
Surgical margins	R0/R1 vs. R2 or no resection	0.0001	2.839	(1.696–4.752)
Age	-	0.022	1.017	(1.003–1.032)
Grade	G2 vs. G3	0.153	1.362	(0.891–2.081)
Biomarker	High SIRT1 and low TOP2A	0.002	2.547	(1.406–4.612)

**Table 5:** Antibodies used for immunohistochemistry.

Antibody	Dilution	Company
SIRT1 HPA006295 Rabbit IgG monoclonal	1:250	Atlas antibodies (Stockholm, Sweden)
TOP2A D10G9 Rabbit IgG monoclonal	1:200	Cell Signaling Tech. (Danvers, MA, USA)

**Table 6:** Semi-quantitative evaluation of immunostaining.

Coloration Intensity	Fraction of Positive Cells
Negative reaction: 0	0–9%
Positive reaction: 1	10–100%

## Data Availability

De-identified data presented in this study are not publicly available but can be requested from the corresponding author.
